# Analysis of Clinical Symptoms and Biochemical Parameters in Odontogenic Cellulitis of the Head and Neck Region in Children

**DOI:** 10.3390/children10010172

**Published:** 2023-01-16

**Authors:** Adrianna Słotwińska-Pawlaczyk, Bogusława Orzechowska-Wylęgała, Katarzyna Latusek, Anna Maria Roszkowska

**Affiliations:** 1 Department of Pediatric Otolaryngology, Head and Neck Surgery, Chairs of Pediatric Surgery, Medical University of Silesia, 40-055 Katowice, Poland; 2 Ophthalmology Clinic, Department of Biomedical Sciences, University Hospital of Messina, 98122 Messina, Italy

**Keywords:** dental caries, odontogenic infection, children, pediatric patients, facial cellulitis, NLR, CRP

## Abstract

Many cases of cellulitis in the head and neck region among hospitalized pediatric patients are related to odontogenic infections. C-reactive protein (CRP), white blood cell (WBC) count, neutrophils to lymphocytes ratio (NLR), D-dimer, and prealbumin can be used to assess the severity of odontogenic inflammation. The aim of the study is to evaluate the biochemical parameters as a predictor factor of the severity of odontogenic cellulitis in children. This study was conducted from 2020 to 2021 on patients admitted to the Department of Pediatric Otolaryngology and Pediatric Head and Neck Surgery of the Upper Silesian Children’s Health Center in Katowice. We included 40 patients aged 2–16 in the study, who were divided into two groups: research (SS-Study subject) (*n* = 20) and control (CS-Control subject) (*n* = 20). The patients underwent an interview and physical examination to assess the presence of intraoral and extraoral swelling and the presence of trismus. The patients who qualified for the study had blood taken to determine the level of CRP, WBCs, NLR, D-dimers, and prealbumin. Differences in biochemical test results in the SS and CS were statistically significant (*p* < 0.05). In the SS group, the mean values of biochemical parameters exceeded the clinical norm. A statistically significant positive relationship was found between CRP and extraoral swelling. The NLR correlates significantly with extraoral swelling and the length of hospitalization. D-dimer statistically correlated with trismus, extraoral swelling, and the number of anatomical spaces involved. The NLR and CRP ratio can be considered a prognostic marker of the course of infection and hospitalization time.

## 1. Introduction

Primary inflammatory focus is a chronic pathomorphological inflammatory lesion, which is a source of bacterial, toxic, allergic, or nervous influence, and because of that, it can cause clinical symptoms of focal disease and maintain lesions in distant organs. Initially, a small carious lesion of a deciduous tooth or permanent tooth, which is left in the oral cavity without any therapeutic intervention, may lead to irreversible pulpitis. The migration of bacteria from the infected pulp through the apical foramen to the periapical tissues causes inflammation of the periapical tissues or submucosal abscesses and then life-threatening phlegmon of the head and neck space, osteomyelitis, meningitis, or cavernous sinus thrombosis [1,2,3]. Cellulitis is one of the most common complications of odontogenic infection. Odontogenic cellulitis is an acute, deep, and diffuse inflammation of the subcutaneous tissue. It spreads through the spaces between tissue cells to tissue spaces and the entire aponeurosis plane due to odontogenic infection [4]. Because of the anatomical structure of the craniofacial region, the inflammatory process most often spreads through the continuity, where the resistance of the tissues is the lowest. The direction of infection spread depends on the topography of the causal tooth root in the bone. Fascial spaces are connected to each other by loose connective tissue along the fascia, blood vessels, and nerve trunks, which promotes the spread of inflammation upwards towards the base of the skull or downwards to the parapharyngeal space and mediastinum. The spread of infection through the blood and lymphatic vessels is relatively rapid in the head and neck area. In the case of blood vessels, veins are more likely to transfer bacteria and their toxins because they have thin walls, no valves in the head area, and slower blood flow, which promotes the formation of an infected clot. It is important to eliminate the cause of inflammation and treat symptoms immediately to prevent the spread of infection through blood or lymphatic vessels to distant organs [5]. It is absolutely necessary to remember the anatomical, pathophysiological, and pharmacokinetic differences between pediatric and adult patients [6,7]. In the group of adults, odontogenic cellulitis of the head and neck is multimicrobial with a significant component of anaerobic bacteria, while in children, the most common causes are staphylococci and streptococci [8]. The cause of odontogenic infections is often endogenous oral bacteria and not introduced non-resident bacteria [9]. Antibiotic therapy should only be used to treat the odontogenic infection when the infection spreads and symptoms such as extraoral swelling or fever appear. The antibiotic of first choice is amoxicillin or amoxicillin with clavulanic acid. In case of allergy to the above antibiotics, it is recommended to use clindamycin, azithromycin, moxifloxacin, or targeted antibiotic [10].

CRP is the major acute phase protein that shows a dynamic and marked increase in serum concentration during infection or as a result of tissue damage. Serum CRP protein is almost absent in healthy individuals but increases up to 1000-fold when tissue damage occurs during infection, tissue trauma, and inflammation. The half-life is about five to seven hours. Prealbumins belong to the group of negative acute phase proteins, whose concentration in the blood serum decreases during the immune response during the inflammatory process. The half-life of prealbumin is 1.9–2.0 days. The relatively short half-life is an advantage of both prealbumin and CRP, which could make them sensitive indicators of infection. The WBC count determines the total number of white blood cells in the blood serum and is a non-specific test. Values above 10,000 leukocytes/mm^3^ may suggest leukocytosis, which may be caused by pathological conditions in the body, such as inflammation, bacterial infections, tissue damage, or cancer, while an elevated WBC count may occur physiologically in humans after physical exercise or eating. The number of neutrophils in the blood serum increases with the severity of the inflammatory disease in the body, while the level of lymphocytes determines the patient’s immunity and decreases with the severity of the inflammation. Therefore, the NLR is a more reliable indicator in the assessment of inflammation than the determination of the total leukocyte count. The assessment of the D-dimer level is mainly used in the diagnosis of deep vein thrombosis, pulmonary embolism, aortic dissection, and disseminated intravascular coagulation (DIC). An elevated D-dimer level is also found in patients with infections and inflammations, injuries, recent surgical operations, liver and kidney diseases, and during pregnancy, but it is less specific in the above conditions. The assessment of these parameters may be helpful in the diagnosis and then in the selection of treatment for odontogenic cellulitis

Some studies confirm that more than 50% of all cellulitis in the head and neck region among hospitalized pediatric patients is related to odontogenic infections [11,12], while more than 50% of all odontogenic cellulitis requires hospitalization treatment [13]. A better understanding of the problem of odontogenic cellulitis and periodically updating one’s knowledge in the field of epidemiology, etiology, symptomatology, and treatment by general dentists, pedodontists, and pediatricians can help make an accurate diagnosis earlier and implement effective treatment faster. The aim of the study is to evaluate the biochemical parameters of CRP, NLR, prealbumin, D-dimer, and WBCs as predictor factors of the severity of an odontogenic connective tissue inflammation of the head and neck region in children.

## 2. Materials and Methods

We conducted a retrospective study and qualified patients with diagnosed odontogenic foci in the oral cavity. The study was conducted at the Department of Pediatric Otolaryngology and Pediatric Head and Neck Surgery of the Upper Silesian Children’s Health Center in Katowice in 2020–2021. We included 40 patients aged 2–16 in the study, who were divided into 2 groups: research (SS-Study subject) (*n =* 20) and control (CS-Control subject) (*n =* 20). The CSs consisted of patients referred for the extraction of potential odontogenic inflammatory foci, which were not affected by an active inflammatory process (for orthodontic reasons: persistent teeth, impacted teeth, supernumerary teeth, additional teeth). The SSs were patients with an active odontogenic inflammatory process in the head and neck area, including teeth with gangrene of the pulp, chronic inflammation of the periapical tissues, as well as abscesses in the head and neck area. The exclusion criteria of patients from the study were systemic diseases, patients with non-odontogenic infections, rhinosinusitis, inflammation of the salivary glands, immunocompromised patients, patients after radiotherapy of the head and neck area, patients after systemic antibiotic therapy in the last 6 weeks, patients undergoing steroid therapy, and lack of consent of the legal guardian for the child’s participation in the study. The patients underwent an interview, physical examination, and imaging diagnostics in order to assess odontogenic foci in the oral cavity. The patients who qualified for the study had 3 mL of peripheral blood taken to determine the level of CRP, NLR, prealbumin, and D-dimers and the number of white blood cells (WBCs). We assessed the presence of intraoral and extraoral swelling and the presence of trismus during the physical examination.

This study was approved by the Ethics Committee of the Medical University of Silesia (Protocol No. KNW/0022/KB1/76/19) and conducted in accordance with the World Medical Association Declaration of Helsinki. The data was analyzed using Statsmodels (Python package) version 0.13.2. Descriptive statistics were prepared for each parameter. The Student’s *t*-test and the Mann–Whitney test were used to analyze the mean values and standard deviations in SS and CS. A linear regression test y = b_1_x + b_0_ was used to determine the correlation of biochemical values with the clinical parameters, such as the length of hospital stay. Spearman’s correlation analysis was used to examine the correlation between the values of diagnostic tests, the length of hospital stay, and the severity of selected clinical symptoms.

## 3. Results

In the SS group, 60% were girls, and 40% were boys, while in the CS group, 75% were boys and 25% were girls. Patients who qualified for the study were in the 2–16 age range. The values of diagnostic tests and the mean, minimum and maximum values for the hospitalization period for SS and CS are presented in Table 1, Table 2 and Table 3. The Student’s *t*-test was used to analyze the results of biochemical tests: CRP, WBCs, NLR, D-dimer, and prealbumin. In the whole group, the mean values of CRP, WBCs, NLR, D-dimer, and prealbumin were 32.9 mg/L; 9.1 × 10^3^/µL; 4.7; 879.4 ng/mL, and 0.2 g/L, respectively, while the mean hospital stay was 2.1 days. In the SS group, the mean value of CRP was 64.2 mg/L, WBC count 11.9 × 10^3^/µL, NLR 7.6, D-dimer 1521 ng/mL, Prealbumin 0.1 g/L and the mean hospitalization time was four days. In the CS group, the mean value of CRP was 1.5 mg/L, WBC count 6.4 × 10^3^/µL, NLR 1.8, D-dimer 237.8 ng/L, Prealbumin 0.2 g/L, and the hospitalization period was one day. The maximum values of CRP, WBCs, NLR, D-dimer, and prealbumin were 166.6 mg/L; 22.6 × 10^3^/µL; 41.1; 12,958 ng/L, and 0.28 g/L, respectively, while the minimum values were 0.0 mg/L; 4.2 × 10^3^/L; 0.6; 14 ng/L, and 0.08 g/L, respectively. The Mann–Whitney test was used to assess the time of hospitalization. The mean hospitalization time for all patients was 2.1, while for SS, it was 4.0, and for CS 1.0.

Spearman’s correlation coefficient showed a statistically significant, positive correlation between CRP and NLR as well as CRP and D-dimer, while the correlation between CRP and the WBC count turned out to be positive but not statistically significant. Prealbumins showed a negative correlation with all biochemical parameters, while the correlation of prealbumins with the WBC count, NLR, and D-dimer was statistically significant. The NLR ratio showed a positive, statistically significant relationship with CRP and also with D-dimers (Table 4).

Spearman’s correlation coefficient was also used to analyze the relationship between biochemical results and clinical parameters, which are presented in Table 5. A statistically significant positive relationship was found between CRP and extraoral swelling, while the correlation between CRP and trismus was negative but not statistically significant. The NLR ratio correlated positively and statistically significantly with extraoral swelling and the length of hospitalization but negatively with trismus. The level of prealbumin showed no statistically significant relationship with any of the examined clinical parameters. However, D-dimer statistically correlated with trismus, extraoral swelling, and the number of anatomical spaces involved (Table 6).

**Figure 1:**
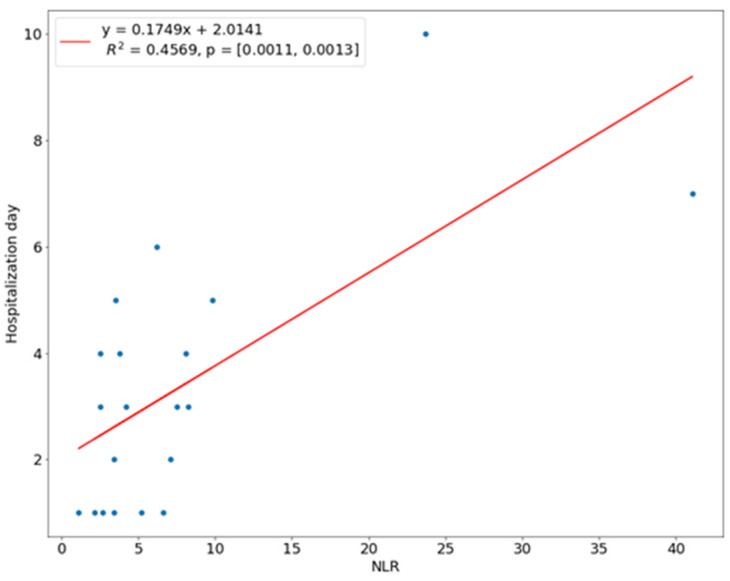
Correlation of NLR with length of hospital stay.

**Figure 2:**
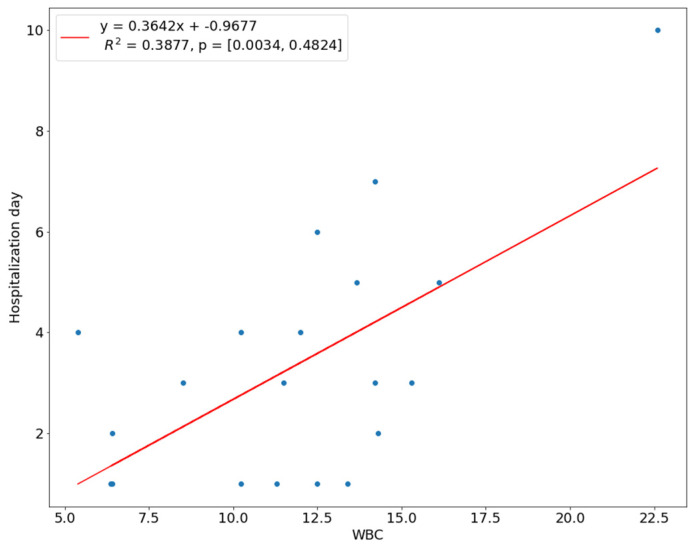
Correlation of WBC count with length of hospital stay.

**Figure 3:**
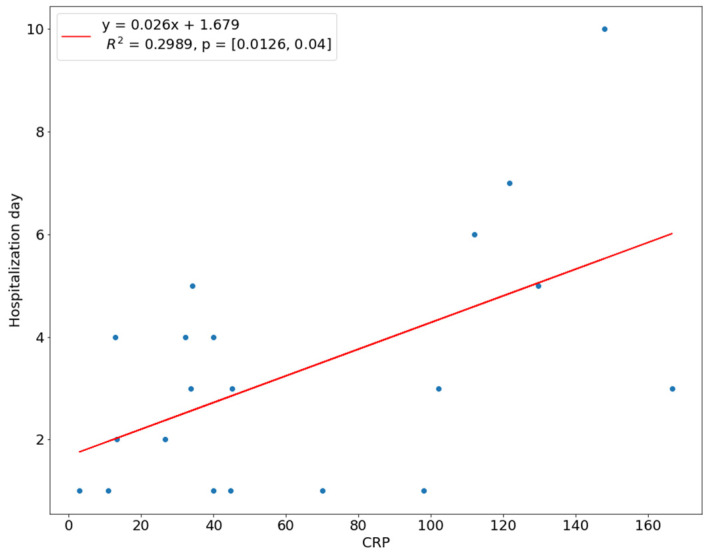
Correlation of CRP with length of hospital stay.

**Figure 4:**
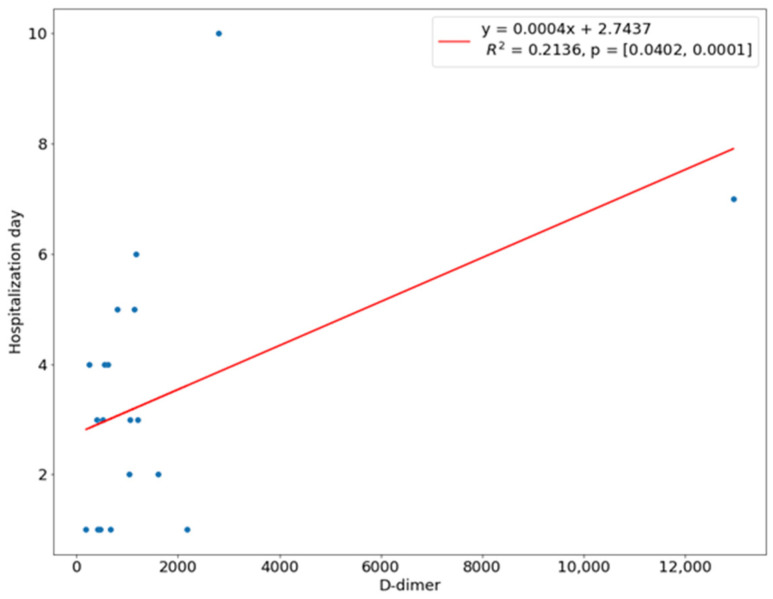
Correlation of D-dimer with length of hospital stay.

We introduced a linear regression model to investigate the relationship between the length of hospital stay and the predictor variables. The NLR ratio was a significant predictor of hospital stay (*p* < 0.01). The coefficient of determination R^2^ for this model was 0.4569, which means 45.69% of the variation in the length of stay could be assigned to the NLR value at admission (Figure 1). There was a linear relationship between the WBC count and CRP with the time of hospital stay, i.e., the higher the WBC count or CRP level, the longer the hospitalization. This linear relationship means that 38.77% of the variation during a hospital stay was explained by the WBC count, while 29.89% of the variation was explained by the amount of CRP (Figure 2 and Figure 3). In 21.36%, the length of hospitalization could be predicted based on the level of D-dimer at hospital admission (Figure 4). The level of prealbumin showed no significant correlation with the length of hospitalization.

## 4. Discussion

It should be emphasized that all the studies mentioned in this article are based on a group of adult patients. We did not find analogous studies in the PubMed database that evaluated biochemical parameters in relation to odontogenic cellulitis of the head and neck region in children. In this study, we analyzed odontogenic infections in children. We compared the course of infections in qualified patients and found a correlation between clinical and laboratory parameters. The results of our analysis indicate that odontogenic infections of the connective tissue of the head and neck in children have a characteristic clinical picture that may be helpful in the treatment of these infections. Dental caries and periapical tissue inflammation have been implicated as causes of dentoalveolar tissue inflammation. Doll et al., in their study, qualified 88.3% with a similar cause and 6.7% with an abscess formed after tooth extraction [14].

Serum CRP protein is almost absent in healthy people but can increase a thousand-fold when tissue is damaged during infection, tissue trauma, or inflammation. The normal value of CRP in the blood serum ranges from 1–10 mg/L, and the half-life is about five to seven hours. It was assumed that high serum CRP concentration at admission was associated with the severity and advanced course of odontogenic infections [15,16,17]. In the study, we assessed that CRP had a high correlation with the severity of infection, based on a statistically significant correlation between CRP level and the size of swelling in patients treated in our hospital (*p* < 0.05). A high correlation was also found between CRP and length of hospitalization (R = 0.3; *p* < 0.05), and similar results were obtained by Sharma et al. [18] (R = 0.4; *p* < 0.01) and Pinilla et al. [19] (R = 0.45, *p* < 0.01). Stathopoulos et al. also confirmed the presence of a positive linear relationship between CRP and hospitalization time [17]. Begue et al. also found a strong correlation between the two variables (Rs = 0.579) and a linear relationship with the R2 value of 0.294 [20]. Heim et al., in a study, assessed that CRP was significant in assessing the severity of orofacial infections based on the time of hospitalization. They found that CRP levels differed statistically between the group of patients hospitalized for ten days or more compared to the group where the duration of hospitalization was seven to nine days (*p* < 0.001) [21]. In this study, we found a statistically significant difference between the mean CRP value of the SS group (64.2 × 10^3^/μL) and CS group (1.5 × 10^3^/μL), where *p* < 0.001, and a similar conclusion was also drawn by Sharma et al. [18] and Ren et al. [22].

The number of leukocytes increases during the immune reaction [23], but the increase in the WBC count is much slower than the increase in CRP [24]. In the study, we describe a statistically significant difference in the amount of the WBC count between the SS group (11.9 × 10^3^/μL) and the CS group (6.4 × 10^3^/μL), where *p* < 0.001. A positive relationship was found between the amount of the WBC count and the appearance of extraoral swelling and a negative relationship with the presence of trismus, while these results have *p* > 0.05. Kaur et al. evaluated that preoperative WBC values showed a significant correlation with the severity of infection (*p* < 0.01) based on a pain assessment and limitations on opening the mouth. Additionally, they proved that both the WBC and CRP levels correlated with the clinical improvement of patients, i.e., with a decrease in the WBC and CRP levels, the symptoms of infection disappear [25]. Bagul et al. reached similar conclusions [26]. Some authors claim that the WBC count at the time of admission to the hospital is a predictor of hospitalization time in patients with odontogenic infections [16], while other authors believe that the WBC count cannot be used to predict the duration of a hospital stay [17,27]. According to Wang et al., the initial WBC count > 15,000 correlated positively with the length of hospital stay, which lasted more than four days. They assessed the WBC count as a useful predictor of hospital length of stay [28]. We found the relationship between the WBC count and hospitalization time based on Spearman’s correlation at the level of *p* = 0.05, and a positive linear regression model was obtained (R = 0.39).

Another analyzed indicator was the value of the NLR, which determines the ratio of the number of neutrophils to lymphocytes, which is assessed on the basis of complete blood count and does not require additional tests. The number of neutrophils in the blood serum increases with the severity of the inflammatory disease in the body, while the level of lymphocytes determines the patient’s immunity and decreases with the severity of the inflammation. Therefore, the NLR is a more reliable indicator in the assessment of inflammation than the determination of the WBC count [29]. We also found a statistically significant difference in the mean NLR in the SS group (7.6) and CS group (1.8), where *p* < 0.01. Dogruel et al. evaluated NLR as an important indicator of recovery from severe odontogenic infections [30]. Gallagher et al. drew similar conclusions and showed the possibility of predicting a time of hospitalization longer than two days based on NLR at a significance level similar to CRP [31]. We found a statistically significant Spearman relationship between NLR and the severity of infection based on the assessment of extraoral edema and limitation on opening the mouth. Additionally, we obtained a positive linear regression model in correlation with the length of hospitalization (R = 0.46, *p* < 0.01).

Prealbumin belongs to the group of negative acute phase proteins, whose concentration in the blood serum decreases during the inflammatory process. The mechanism of the decrease in the concentration of prealbumin in the blood serum during acute and chronic inflammation is the change in the priorities of protein synthesis in the liver and, as a result, the production of positive acute phase proteins increases and the production of negative acute phase proteins decreases [32]. Low levels of prealbumin may be the result of anorexia and malnutrition associated with trismus, pain, discomfort due to swelling, dysphagia, or discharge. The half-life of prealbumin is 1.9–2.0 days [33]. The relatively short half-life is an advantage of both prealbumin and CRP, which makes them considered sensitive indicators of infection. The relatively short half-life is an advantage of both prealbumin and CRP proteins; therefore, they are sensitive indicators of infection. In this study, the mean prealbumin concentration was significantly different between the SS group (0.1 g/L) and the CS group (0.2 g/L), where *p* < 0.001. Adeosun et al. obtained similar results when they compared the biochemical results of outpatients and inpatients. He also found a significantly negative correlation between the level of prealbumin and the number of involved deep anatomical spaces and hospitalization time [34]. Sharma et al. and Cunningham et al. determined prealbumin values significantly lower than normal concentrations in patients admitted for treatment. They confirmed the relationship between the concentration of prealbumin and the length of stay in the hospital (*p* < 0.01), the higher the level of prealbumin, the shorter the time in the hospital [18,33]. In our study, we found a significant relationship between the level of prealbumin and other biochemical parameters—the WBC count, NLR, and D-dimer—while the negative correlation with extraoral edema, the number of involved anatomical spaces, and the duration of hospitalization did not turn out to be statistically significant.

D-dimer molecules are a product of the fibrinolysis process. The presence of D-dimers in the blood serum indicates intravascular coagulation; therefore, they are an indicator of the activation of the coagulation process. Nowadays, the assessment of D-dimer levels is mainly used in the diagnostics of deep venous thrombosis, pulmonary thromboembolism, dissection of the aorta, and disseminated intravascular coagulation (DIC). Higher levels of D-dimer are also found in patients with infections and inflammations, injuries, or after a recent surgical operation. Higher levels of D-dimers are less specific in the above conditions [35]. No articles were found in the PubMed database that directly describe the relationship between D-dimer levels and odontogenic inflammation, but we found a study that shows a probable relationship between D-dimer levels and periodontal disease [36]. During bacterial colonization in periodontal disease, the host immune system can cause tissue damage, which activates the humoral immune response [37]. Levi et al. say that inflammation leads to the activation of coagulation, and coagulation significantly affects inflammatory activity [38]. In this study, there was a significant difference in the level of D-dimer in the SS group (1521 ng/mL) and the CS group (237.8 ng/mL), as well as a significant positive correlation between the level of D-dimer and CRP and the NLR ratio and significant negative correlation with prealbumin. In 21.36%, the length of hospitalization could be predicted on the basis of D-dimer level on the day of admission to the hospital.

A limitation of the present study is that its retrospective nature limited the possibility of drawing more characteristic conclusions. Another limitation of this study is that the number of patients is not very large. The severity of the infection and the length of hospital stay are influenced by many complex factors. We evaluated several parameters, and therefore, further studies with larger groups of patients and additional measurements of biochemical parameters at regular intervals should be carried out to be able to use markers of inflammation to predict the course of infection.

## 5. Conclusions

CRP and NLR are traditional diagnostic tests and can reliably predict the clinical course of odontogenic infection. Additionally, the NLR can be considered not only a significant prognostic marker of the course of infection but also of hospitalization time because it shows a significant correlation between the time of hospitalization and general clinical symptoms. The amount of the WBC count helps determine the patient’s condition at admission but cannot be considered a sensitive prognostic indicator. Serum prealbumin and D-dimer may be a measure of disease severity of odontogenic cellulitis of the head and neck region in children. More studies are needed to validate these data in a pediatric population. It is also recommended to extend the study with control visits to assess the regression of the biochemical parameters tested to confirm their significance in the assessment of treatment effectiveness.

## Figures and Tables

**Table 1:** Biochemical parameters characteristics of study subjects (SS).

Number	CRP	WBC Count	D-Dimer	Prealbumin	NLR Ratio
1	32.2	5.39	618	0.14	2.5
2	166.60	11.5	1051	0.13	7.5
3	112	12.5	1167	0.15	6.2
4	129.6	13.66	1138	0.09	9.8
5	26.7	14.3	1038	0.08	3.4
6	102.2	14.2	1207	0.09	8.25
7	3	6.4	417	0.16	1.1
8	12.9	12	540	0.13	8.1
9	13.3	6.4	1599	0.11	7.1
10	44.7	10.24	2180	0.12	6.6
11	40	10.22	246	0.14	3.8
12	33.8	15.3	390	0.13	4.2
13	45.1	8.5	511	0.15	2.5
14	11	6.37	179	0.15	2.7
15	34.1	16.1	795	0.1	3.5
16	40	13.4	466	0.1	3.4
17	147.9	22.6	2797	0.13	23.7
18	121.6	14.2	12,958	0.13	41.1
19	98	11.3	453	0.15	5.2
20	70	12.5	671	0.14	2.15

**Table 2:** Biochemical parameters characteristics of control subjects (CS).

Number	CRP	WBC Count	D-Dimer	Prealbumin	NRL Ratio
1	2.2	8.52	544	0.26	1.7
2	2.4	9.3	560	0.25	2.1
3	3.1	9.69	98	0.24	2.5
4	1.9	9.32	64	0.18	0.6
5	0.4	4.2	14	0.2	1.5
6	4.7	6.58	35	0.2	1
7	0	5.9	47	0.26	1.3
8	3.4	6.46	466	0.21	0.8
9	0.7	8.56	21	0.16	3.5
10	0.3	4.83	167	0.26	1.5
11	0.3	5.99	199	0.23	1
12	1.6	6	680	0.26	1.9
13	2.5	4.2	182	0.18	1.2
14	0	5.9	359	0.21	1.7
15	0.6	4.23	319	0.28	1.3
16	0.9	5.8	189	0.19	2.6
17	0.7	5.5	158	0.24	2.6
18	1.1	6.1	236	0.19	2.9
19	0.3	4.7	141	0.18	1.8
20	3.4	6.7	278	0.2	2.0

**Table 3:** Mean value analysis of biochemical parameters.

Parameter	SS + CS	SS	CS	*p* Value
CRP	32.9 ± 46.7	64.2 ± 49.0	1.5 ± 1.3	<0.001
WBC Count	9.1 ±4.1	11.9 ± 4.0	6.4 ± 1.7	<0.001
NLR	4.7 ± 7.0	7.6 ± 9.0	1.8 ± 0.7	0.007
D-dimer	879.4 ± 2019.3	1521.0 ± 2701.1	237.8 ± 189.8	0.046
Prealbumin	0.2 ± 0.1	0.1 ± 0.0	0.2 ± 0.0	<0.001
Hospitalization	2.1 ± 2.1	4.0 ± 2.6	1.0 ± 0.0	<0.001

**Table 4:** Correlation between diagnostic biomarkers.

Parameter	CRP	WBC	NLR	D-dimer	Prealbumin
CRP	1.00				
WBC Count	0.43	1.00			
NLR	0.57 *	0.45	1.00		
D-dimer	0.55 *	0.39	0.67 *	1.00	
Prealbumin	−0.14	−0.58 *	−0.45 *	−0.49 *	1.00

* *p* < 0.05 is considered to be statistically significant.

**Table 5:** Clinical parameters characteristics of study subjects.

Number	Trismus [mm]	Extraoral Swelling [mm]	Intraoral Swelling	Length of Hospitalization	Number of Anatomic Spaces Involved
1	25	12	+	4	2
2	19	24	+	3	2
3	−	12	+	6	2
4	22	22	+	5	1
5	−	10	+	2	2
6	27	19	+	3	3
7	29	14	+	1	1
8	20	17	+	4	2
9	−	16	+	2	1
10	24	20	+	1	3
11	−	0	−	4	0
12	−	0	−	3	0
13	−	0	+	3	2
14	−	0	+	1	2
15	28	18	+	5	1
16	−	0	+	1	1
17	22	26	+	10	3
18	23	23	+	7	3
19	25	20	+	1	1
20	−	0	+	1	2

**Table 6:** Correlation between diagnostic biomarkers and clinical parameters.

Parameter	Trismus	Extraoral Swelling	Length of Hospitalization	Number of Anatomic Spaces Involved
CRP	−0.43	0.56 *	0.44 (*p =* 0.054)	0.36
WBC Count	−0.16	0.29	0.44 (*p =* 0.05)	0.12
NLR	−0.63 *	0.76 *	0.56 *	0.34
D-dimer	−0.44 *	0.7 *	0.44 (0.055)	0.64 *
Prealbumin	0.19	−0.29	−0.15	−0.08

* *p* < 0.05 is considered to be statistically significant.

## Data Availability

Data is contained within this article [Table 1, Table 2, Table 3 and Table 5].

## References

[1] Nair P.N. (2004). Pathogenesis of apical periodontitis and the causes of endodontic failures. Crit. Rev. Oral Biol. Med..

[2] Bali R.K., Sharma P., Gaba S., Kaur A., Ghanghas P. (2015). A review of complications of odontogenic infections. Natl. J. Maxillofac. Surg..

[3] Han X., An J., Zhang Y., Gong X., He Y. (2016). Risk Factors for Life-Threatening Complications of Maxillofacial Space Infection. J. Craniofac. Surg..

[4] Giunta Crescente C., Soto de Facchin M., Acevedo Rodríguez A.M. (2018). Medical-dental considerations in the care of children with facial cellulitis of odontogenic origin. A disease of interest for pediatricians and pediatric dentists. Arch. Argent Pediatr..

[5] Biasotto M., Pellis T., Cadenaro M., Bevilacqua L., Berlot G., Di Lenarda R. (2004). Odontogenic infections and descending necrotising mediastinitis: Case report and review of the literature. Int. Dent. J..

[6] Gonzalez L.P., Pignaton W., Kusano P.S., Módolo N.S., Braz J.R., Braz L.G. (2012). Anesthesia-related mortality in pediatric patients: A systematic review. Clinics.

[7] da Fonseca M.A., Nelson T., Wright G.Z., Kupietzky A. (2014). The use of general anesthesia in behavior management. Behavior Management in Dentistry for Children.

[8] Perina V., Szaraz D., Harazim H., Urik M., Klabusayova E. (2022). Paediatric Deep Neck Infection-The Risk of Needing Intensive Care. Children.

[9] Orzechowska-Wylęgała B., Wylęgała A., Buliński M., Niedzielska I., Madej A. (2017). Pharmacoeconomic analysis of antibiotic therapy in maxillofacial surgery. BDJ Open..

[10] Wylęgała A., Paluch M., Orzechowska-Wylęgała B., Galicka-Brzezina A., Chyrek K., Madej A. (2019). Pharmacoeconomic analysis of antibiotic therapy in surgical site infections. Int. J. Clin. Pharmacol. Ther..

[11] Unkel J.H., McKibben D.H., Fenton S.J., Nazif M.M., Moursi A., Schuit K. (1997). Comparison of odontogenic and nonodontogenic facial cellulitis in a pediatric hospital population. Pediatr. Dent..

[12] Biederman G.R., Dodson T.B. (1994). Epidemiologic review of facial infections in hospitalized pediatric patients. J. Oral Maxillofac. Surg..

[13] Lin Y.T., Lu P.W. (2006). Retrospective study of pediatric facial cellulitis of odontogenic origin. Pediatr. Infect. Dis. J..

[14] Doll C., Carl F., Neumann K., Voss J.O., Hartwig S., Waluga R., Heiland M., Raguse J.D. (2018). Odontogenic Abscess-Related Emergency Hospital Admissions: A Retrospective Data Analysis of 120 Children and Young People Requiring Surgical Drainage. Biomed. Res. Int..

[15] Pepys M.B., Booth S.E., Tennent G.A., Butler P.J., Williams D.G. (1994). Binding of pentraxins to different nuclear structures: C-reactive protein binds to small nuclear ribonucleoprotein particles, serum amyloid P component binds to chromatin and nucleoli. Clin. Exp. Immunol..

[16] Ylijoki S., Suuronen R., Jousimies-Somer H., Meurman J.H., Lindqvist C. (2001). Differences between patients with or without the need for intensive care due to severe odontogenic infections. J. Oral Maxillofac. Surg..

[17] Stathopoulos P., Igoumenakis D., Shuttleworth J., Smith W., Ameerally P. (2017). Predictive factors of hospital stay in patients with odontogenic maxillofacial infections: The role of C-reactive protein. Br. J. Oral Maxillofac. Surg..

[18] Sharma A., Giraddi G., Krishnan G., Shahi A.K. (2014). Efficacy of Serum Prealbumin and CRP Levels as Monitoring Tools for Patients with Fascial Space Infections of Odontogenic Origin: A Clinicobiochemical Study. J. Maxillofac. Oral Surg..

[19] Pinilla J.C., Hayes P., Laverty W., Arnold C., Laxdal V. (1998). The C-reactive protein to prealbumin ratio correlates with the severity of multiple organ dysfunction. Surgery.

[20] Bègue L., Schlund M., Raoul G., Ferri J., Lauwers L., Nicot R. (2022). Biological factors predicting the length of hospital stay in odontogenic cellulitis. J. Stomatol. Oral Maxillofac. Surg..

[21] Heim N., Wiedemeyer V., Reich R.H., Martini M. (2018). The role of C-reactive protein and white blood cell count in the prediction of length of stay in hospital and severity of odontogenic abscess. J. Craniomaxillofac. Surg..

[22] Ren Y.F., Malmstrom H.S. (2007). Rapid quantitative determination of C-reactive protein at chair side in dental emergency patients. Oral Surg. Oral Med. Oral Pathol. Oral Radiol. Endod..

[23] Aminzadeh Z., Parsa E. (2011). Relationship between Age and Peripheral White Blood Cell Count in Patients with Sepsis. Int. J. Prev. Med..

[24] Boucher N.E., Hanrahan J.J., Kihara F.Y. (1967). Occurrence of C-reactive protein in oral disease. J. Dent. Res..

[25] Kaur A., Sandhu A., Kaur T., Bhullar R.S., Dhawan A., Kaur J. (2019). Correlation Between Clinical Course and Biochemical Analysis in Odontogenic Space Infections. J. Maxillofac. Oral Surg..

[26] Bagul R., Chandan S., Sane V.D., Patil S., Yadav D. (2017). Comparative Evaluation of C-Reactive Protein and WBC Count in Fascial Space Infections of Odontogenic Origin. J. Maxillofac. Oral Surg..

[27] Peters E.S., Fong B., Wormuth D.W., Sonis S.T. (1996). Risk factors affecting hospital length of stay in patients with odontogenic maxillofacial infections. J. Oral Maxillofac. Surg..

[28] Wang J., Ahani A., Pogrel M.A. (2005). A five-year retrospective study of odontogenic maxillofacial infections in a large urban public hospital. Int. J. Oral Maxillofac. Surg..

[29] Huang Z., Fu Z., Huang W., Huang K. (2020). Prognostic value of neutrophil-to-lymphocyte ratio in sepsis: A meta-analysis. Am. J. Emerg. Med..

[30] Dogruel F., Gonen Z.B., Gunay-Canpolat D., Zararsiz G., Alkan A. (2017). The Neutrophil-to-Lymphocyte ratio as a marker of recovery status in patients with severe dental infection. Med. Oral Patol. Oral Cir. Bucal..

[31] Gallagher N., Collyer J., Bowe C.M. (2021). Neutrophil to lymphocyte ratio as a prognostic marker of deep neck space infections secondary to odontogenic infection. Br. J. Oral Maxillofac. Surg..

[32] Evans D.C., Corkins M.R., Malone A., Miller S., Mogensen K.M., Guenter P., Jensen G.L., the ASPEN Malnutrition Committee (2021). The Use of Visceral Proteins as Nutrition Markers: An ASPEN Position Paper. Nutr. Clin. Pract..

[33] Cunningham L.L., Madsen M.J., Van Sickels J.E. (2006). Using prealbumin as an inflammatory marker for patients with deep space infections of odontogenic origin. J. Oral Maxillofac. Surg..

[34] Adeosun P.O., Fatusi O.A., Adedeji T.A. (2019). Assessment of Severity of Illness and Monitoring Response to Treatment of Odontogenic Space Infection Using Serum Prealbumin. J. Maxillofac. Oral Surg..

[35] Johnson E.D., Schell J.C., Rodgers G.M. (2019). The D-dimer assay. Am. J. Hematol..

[36] Sánchez-Siles M., Rosa-Salazar V., Salazar-Sánchez N., Camacho-Alonso F. (2015). Periodontal disease as a risk factor of recurrence of venous thromboembolic disease: A prospective study. Acta Odontol. Scand..

[37] Ramseier C.A., Kinney J.S., Herr A., Braun T., Sugai J.V., Shelburne C.A., Rayburn L.A., Tran H.M., Singh A.K., Giannobile W.V. (2009). Identification of pathogen and host-response markers correlated with periodontal disease. J. Periodontol..

[38] Levi M., van der Poll T., Büller H.R. (2004). Bidirectional relation between inflammation and coagulation. Circulation.

